# A Novel Urine Test Biosensor Platform for Early Lung Cancer Detection

**DOI:** 10.3390/bios13060627

**Published:** 2023-06-06

**Authors:** Ory Wiesel, Sook-Whan Sung, Amit Katz, Raya Leibowitz, Jair Bar, Iris Kamer, Itay Berger, Inbal Nir-Ziv, Michal Mark Danieli

**Affiliations:** 1 Division of Thoracic and Esophageal Surgery the Cardiovascular Center, Tzafon Medical Center, Affiliated to Azrieli Faculty of Medicine, Bar-Ilan University, Poriya 1520800, Israel; owiesel@tzmc.gov.il; 2 Department of Thoracic and Cardiovascular Surgery, Ewha Womens University Seoul Hospital, 260 Gonghang-daero, Gangseo-gu, Seoul 07804, Republic of Korea; swsung@snu.ac.kr; 3 Head of Thoracic Surgery, Rambam Health Care Campus, P.O. Box 9602, Haifa 3109601, Israel; a_katz@rambam.health.gov.il; 4 Oncology institute, Shamir Medical Center, Zerifin 703001, Israel; rayal@shamir.gov.il; 5 Institute of Oncology, Sheba Medical Center, Tel Hashomer, Ramat Gan 5262000, Israel; jair.bar@sheba.health.gov.il (J.B.);; 6 Early OM, 4 Meir Ariel St., Natanya 4253063, Israel; itay.berger@earlylabs.io (I.B.); inbal@earlylabs.io (I.N.-Z.)

**Keywords:** lung cancer, early diagnosis, screening, early detection, volatile organic compound, biosensors, biomarkers

## Abstract

Lung cancer is the leading cause of cancer-related mortality worldwide. Early detection is essential to achieving a better outcome and prognosis. Volatile organic compounds (VOCs) reflect alterations in the pathophysiology and body metabolism processes, as shown in various types of cancers. The biosensor platform (BSP) urine test uses animals’ unique, proficient, and accurate ability to scent lung cancer VOCs. The BSP is a testing platform for the binary (negative/positive) recognition of the signature VOCs of lung cancer by trained and qualified Long–Evans rats as biosensors (BSs). The results of the current double-blind study show high accuracy in lung cancer VOC recognition, with 93% sensitivity and 91% specificity. The BSP test is safe, rapid, objective and can be performed repetitively, enabling periodic cancer monitoring as well as an aid to existing diagnostic methods. The future implementation of such urine tests as routine screening and monitoring tools has the potential to significantly increase detection rate as well as curability rates with lower healthcare expenditure. This paper offers a first instructive clinical platform utilizing VOC’s in urine for detection of lung cancer using the innovative BSP to deal with the pressing need for an early lung cancer detection test tool.

## 1. Introduction

Lung cancer is the leading cause of cancer-related mortality worldwide and is undoubtedly one of the most serious public health concerns of the last two centuries [1,2,3,4,5,6]. Projections published by the American Cancer Society, Inc., report an estimate of 127,070 deaths from lung cancer in the US for 2023 [7]. These numbers reflect a gloomy picture of our current ability to treat this condition. A 2021 report from the American Cancer Society (ACS) states that most lung cancer cases in the United States are diagnosed in later stages, which mandates aggressive, costly treatments with projected five-year survival of less than 20% [8,9].

Lung cancer is broadly divided into small-cell (SCLC, approx. 15% cases) and non-small-cell lung cancer (NSCLC, approx. 85% cases). The main histological subtypes of NSCLC are adenocarcinoma and squamous cell carcinoma [10]. If identified at an early stage, surgical resection of NSCLC offers a favorable prognosis, with 5-year survival rates of 70–90% for small, localized tumors (stage I) [11,12]. Early detection and surgical intervention can contribute to 5-year life extension in about 50% of cases [13,14].

The two components of early detection of cancer are early diagnosis and screening. Early diagnosis focuses on detecting cancer with symptomatic patients as early as possible, while screening consists of testing healthy individuals to identify those having cancer before any symptoms appear [15]. The current lung cancer screening programs are based on the National Lung Cancer Screening Trial (NLST) using low-dose computed tomography scans (LDCT) [16]. While LDCT lung cancer screening programs exist, these tests are associated with overdiagnosis and overtreatment, disparities in adherence, low positive predictive value (PPV: proportion of true positives among those with a positive test result) as well as a high cumulative false-positive rate when used sequentially (low PPV) [14]. Furthermore, the long elapsing time between referral to test, duration of the test itself, and test inconvenience, all contribute to the low test utilization within the eligible population—which was only 5% in 2020 [17]. Therefore, there is still a great unmet need for developing a novel, rapid, simple, and reliable lung cancer detection test [18]. The latter will facilitate diagnosis by the physician, in combination with traditional imaging techniques, and potentially serve as an alternative or adjunct screening test in the future. Moreover, early detection of cancer will enable treatment with a lower level of toxicity, along with decreased exposure to radiation. Furthermore, early detection of lung cancer in early stages is associated with improved overall survival and reduces treatment cost and treatment complexity [13,14,19,20]. It is well recognized that tumors and tumor cells secrete compounds into the bloodstream and subsequently to the urine so that these may be detected using in vitro diagnostic testing [21]. The aqueous urinary matrix contains a small percentage of volatile VOCs. VOCs are a diverse group of carbon-based chemicals that are classified on the basis of their chromatographic retention time and boiling point (ranging from 50 °C to 260 °C) [22]. Urinary metabolomics studies have been employed to different cancer types, such as breast, colorectal, esophageal, pancreatic, prostate, and liver cancers [23]. These molecules, which can be perceived as odors (especially by animals), have been shown to function as “odor signatures” that convey social, emotional, and health information to other members of the species. Studies have shown that lung cancer cells can release specific VOCs in vitro [24]. In addition, the presence of a growing tumor could also induce specific metabolic or nutritional changes in the tumor microenvironment that could alter the production or release of such compounds [22,23,25,26]. Since there is no standardized approach to analyze VOCs, a plethora of techniques and matrices/cell lines were explored, which is reflected in the various VOCs identified. However, comparing VOCs in the headspace of urine, blood, and pleural effusions from patients and lung cancer cell lines showed some overlapping VOCs, indicating their potential use as biomarkers for lung cancer [27].

A novel approach for detection of VOCs from biological samples utilizes the extremely sensitive olfactory abilities of certain animals to detect VOCs and, as a result, to provide an indication of certain biological pathologies, such as lung cancer [22,23]. One of animals’ strongest senses is their ability to smell. A rat is one of the 10 animals with the best sense of smell in nature, as well as an African elephant, a shark, and a grizzly bear. There are various reports about different animals that have been able to be trained in the laboratory to detect VOC’s such as dogs, C. elegans, ants, and birds. We chose Long–Evens type rats because of their operational simplicity, low maintenance costs, lack of dependency on a specific trainer, the animal’s lifespan, and its ability to perform as in integral part of an automatic platform [28,29,30]. According to the International Union of Pure and Applied Chemistry (IUPAC), biosensors (BSs) are considered devices that transform biochemical information into an analytically useful signal. For a timely evaluation of a specific pathological health condition, the BSs need: (i) accurate measurement, (ii) rapid assessment, and (iii) selective detection. These properties of the sensor enables the physician to accurately diagnose the specific disease and initiate required therapy to prevent further aggravation of the disease [23]. Urine tests are an established method for detecting several diseases noninvasively.

In this manuscript, we present a detection system that utilizes the extremely sensitive abilities of Long–Evans rats to detect VOCs from a urine sample to provide an indication of a specific biological pathology, namely lung cancer.

The goal of this work is to present the validation of a BSP. The BSP is a testing platform for recognition of the signature VOCs of lung cancer using trained rats as the BSs. Currently, the test makes no distinction between lung cancer subtypes or their staging. The use of rats as animal BSs in the current BSP has been developed based on data indicating (1) their highly developed sense of smell, which is expressed in their ability to detect odors in very low concentrations; (2) their ability to focus and distinguish a particular odor from within a collection of odors; (3) their ability to learn the desired response to a specific odor; and (4) extensive experience in behavioral work, academically and empirically. The BSP is based on the BS’s (i.e., the trained rats’) sensitive recognition of the unique metabolic signature, composed of VOCs, from a lung cancer patient’s urine sample. The test is intended to serve as a differential diagnosis tool in conjunction with standard diagnostics enabling the physician to proceed with optimal patient management without unnecessary delays or disruption to the existing standard of care, and in the future as a potential tool for routine cancer screening.

## 2. Materials and Methods

### 2.1. Animal Characteristics

Long–Evans rats were inbred and raised at the Early Labs facility. Eighteen male rats were trained in this study. To be included in the study, rats had to be clinically healthy, regularly available for training and familiar with training and testing of odor discrimination procedures (see details below).

### 2.2. Animal Habitat, Nutrition, and Welfare

Animal welfare is an important aspect for the company and conforms with all Welfare Quality^®^ protocols as defined by the American Veterinary Medical Association.

The animals were held in a ventilated room inside a net cage. The room temperature was set to 24 ± 2 °C. The cage dimensions were W180 cm × H210 cm × D180 cm. Each cage held 7–10 rats. The cages included Sani Chip sawdust as a bed, shredded paper, and/or fabric for nesting and covering. The animals were fed with a mixture that contained two types of food with a 1:10 ratio: 1 kg chow for sniff-type rodents and 10 kg of chow for DIETUS 2018sc-type rodents. The dumplings provided were about 10% of the animal’s weight (20–30 gr) for 24 h, etc.; 300–600 mL water bottles were attached to the cage and the animals had ad libitum access to water. The rats’ weight was recorded once a week and every day during gestation. Each day, the food was weighed and divided into boxes for the whole day and the water bottles were emptied and refilled. The food was spread throughout their living facility and water bottles were cleaned every day. The cages were examined daily for shortcomings. The cages were cleaned daily, including their furniture, cage floor, and restroom tray and most of the sawdust was replaced daily. The animals were examined daily by lab professionals for injuries, bleeding, and checked monthly by a veterinarian. Extra emphasis was put on the tidiness of the cage and environmental enrichment. Animals were exposed to different smells, noises, beds, toys, and changes of objects in order to decrease stress. In accordance with European legislation (Dir. 2010/63/EU), no animal was exposed to harmful conditions throughout this study.

### 2.3. The BS’s Training, Validation, and Qualification Process

**Figure 1:**
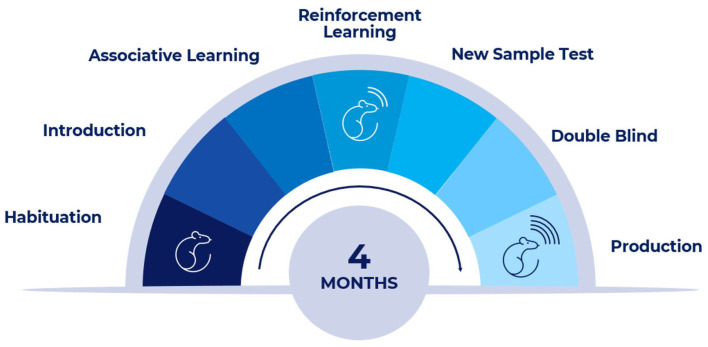
The BS’s training, validation, and qualification process.

Figure 1 describes the training, validation, and qualification steps of the rats included in the study. The steps comprise basic training, BSP sessions (designed familiarize the BS with the technological environment), binary sessions, and repeated internal training. The validation and qualification steps are described in Section 3.3. At each stage, there is a mandatory threshold beyond which the next stage is initiated. 

A multiphase protocol for training Long–Evans rats using an automatic platform was developed. The training sessions are based on behavioral shape methodology and include several consecutive phases at each described step. Human interaction with the platform begins with a habitat for human touch. This can reduce interaction stress, making it easier to detect desired odors. Next, rodents are introduced to the platform environment and learn how to operate within. The following steps include associated learning and detection reporting. Once they have passed these steps with high performance, they can repeat the action in response to the stimulus. Each training session contains between 35 and 60 separate and consecutive exposures to different urine samples in a predefined sequence. 

All exposures are performed at the front of the pod where a tube with urine sample is presented to the rat. The rat pokes its head in the sniffing shaft while the tube is placed in a tube holder. The rat is trained to indicate the positive samples in one area of the pod and the negative sample in another area. For every “correct” answer (i.e., recognizing a pathological sample as such, or a benign sample as such) the biosensor is rewarded with “positive feedback” (i.e., a pellet). The answer is automatically reported to the software (SW). There are no “negative feedbacks” for wrong answers. Thus, the biosensor must make a one-out-of-two decision, a positive or negative answer. After the rat has performed a total of the required correct indications, positive and negative per training session, it can proceed to the next training stages.

Once the animal has passed all training stages, it can move to the final test that is divided into two phases. The first includes testing samples that were used throughout the training sessions. In the second phase, the rat is exposed to a sequence of unknown samples in a double-blind test design: the test is conducted while the animal and the BSP operator are both blinded to the result. Each animal works individually in an isolated pod, so they cannot influence one another and no mimicry effect can occur. The BSs are trained to provide binary assessment of a sample.

### 2.4. The BSP as a Binary Test for Lung Cancer Detection

#### 2.4.1. The System General Overview

**Figure 2:**
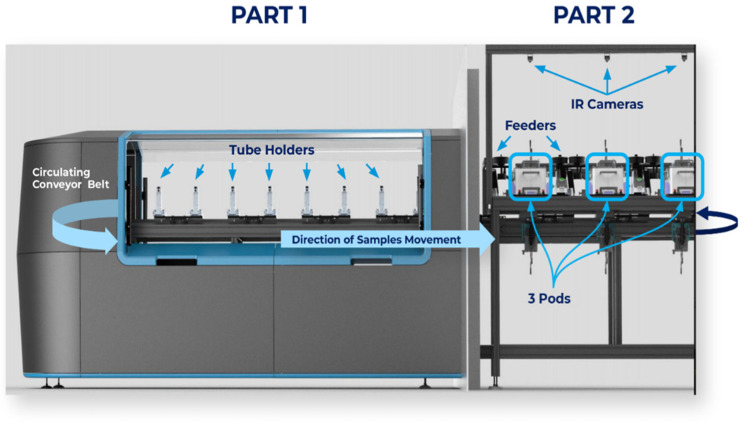
The BSP system, divided into two main parts. Part one (1)—the “operational area”; part two (2)—the “testing area”.

**Figure 3:**
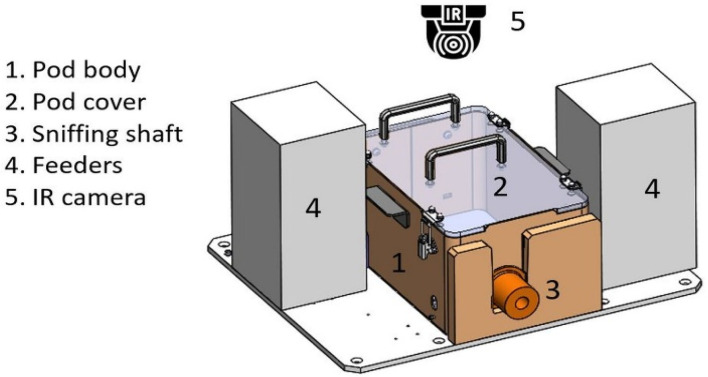
Pod illustration including a pod body (1), pod cover (2), sniffing shaft (3), feeders (4), and IR camera (5). Animals can enter the shaft (3) and smell through the tube when the shaft is open. As soon as the time is up, it closes, and the BS cannot enter. Feeders (4). IR camera (5) senses whether the BS is inside the shaft or not and reports directly to the cloud.

The BSP combines BSs (rats), mechanical components, hardware, software, and a cloud-based database infrastructure (with compliance to data protection). The system is divided into two main parts, as shown in Figure 2. Part one—the “operational area” (Figure 2, left side)—is where the sample tubes are loaded. A conveyor belt with fixed sample holders conveys the urine samples to the BSs’ cages and back. The tube holders are designed to hold a single test tube. The holders keep the open test tube containing the urine sample isolated from the environment except for a hole set to be attached to the exact front location of the sniffing shaft (see explanation below). Part two—(Figure 2, right side) the “testing area”—consists of 3 fixed identical pods (see Figure 3) for 3 separate and independent BSs. 

#### 2.4.2. Pods or “Sniffing Positions”

Each pod holds a BS while it assesses the urine samples through a sniffing shaft. Each BS can assess a single sample at a time, and up to three samples can be assessed simultaneously by the platform. Each pod console comprises the pod body, two feeders, and a sensor wall.

The duration of stay of the animal in the sniffing shaft is monitored by a sensor connected to a timer that counts the number of seconds in which the animal’s head is still inside the sniffing shaft. Once the sample has been tested, the ventilation is turned on to remove the VOC residue, and the sample moves to the next pod to be tested. Each sample is tested three times by three separate and independent BSs.

### 2.5. Sample Preparation and Processing

For the training, validation, and in the final test, urine samples were collected by the patient into a sterile container and were aliquoted into 10 mL sterile polypropylene vacuum tubes. The samples were frozen at −20 °C within 6 h of collection.

The urine samples are stored at −20 °C. The preparation of the samples for the test is performed under the hood. Before placing the sample tube in the system, the urine is aliquoted into 0.5 mL samples and preheated to 60 °C.

A group of 3 BSs assesses each sample in 3 different pods. The response is analyzed using the software algorithm, which provides one output of ± for each individual urine sample (the calculation is explained in Section 2.7). If one of the BSs does not place its nose into the shaft (t = 0) an error is recorded as the output and then a new BS assesses the specific sample. The door closes automatically after 5 s; then, the entire space is automatically aerated through massive ventilation, and the system is ready for the next sample. 

### 2.6. Training Learning Curve

The BSs, which are Long-Evans’s type rats, were inbred and raised at Early Labs. The mothers were exposed to lung cancer VOCs odor prior to their preparation for gestation, and throughout the entire pregnancy, their ability to detect a certain odor increased. The newborn rats (F1 generation) were exposed to lung cancer VOCs from their embryonic phase, infancy phase, and onwards. Training of the F1 generation was conducted between September and July 2021. Training is composed of 7 substages.

**Figure 4:**
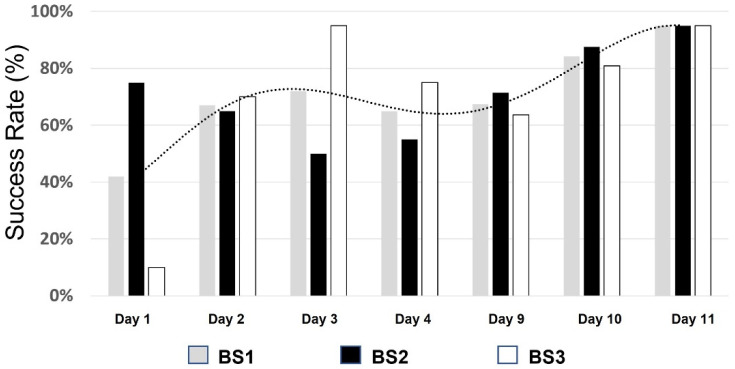
Learning curve of 3 BSs at stage 5 of the training period.

During the training, we analyzed and measured various performance parameters including NPV, PPV, sensitivity, and specificity to create and maintain the biosensor performance standard. If the animal did not reach the required standard, it would not be allowed to proceed to the next phase in the training protocol. This ensures that only animals that are properly trained and meet the criteria are allowed to participate in the testing, and while performance variations do occur, all are within the predefined standard. An example of the advancement of a BS during their training is shown in Figure 4. As can be seen, the success rate of each BS increases as sessions are repeated daily. A clear increase trend in each of the BSs’ performances is seen over time (dotted line). 

During the training process, the best performing rats were selected. Between 7 and 10% did not continue the training program due to insufficient performance levels.

### 2.7. System Software Final Sample Result Calculation

There are two major software (SW) components: the cloud infrastructure and the controller’s embedded software. The software supports the platform, from the first interaction with the animals, including all data related to the animals, through monitoring the samples while interacting with the platform and collecting data points from the platform and the results.

Input parameters of the three BSs are sent to the SW. There, an algorithm calculates the final sample test result, taking into account all 3 BS session, historical performance, and success rates (as well as additional measured parameters).

## 3. Results

### 3.1. A Double-Blind Study for the Assessment of BSP Final Performance 

After the training period, following the completion of all training stages with a characterized performance accuracy, we reached a validation stage in the setting of a clinical trial that includes double-blind test samples. The process of receiving the answer is blinded to the real results. The animals were exposed to a sequence of samples representing the typical observed lung cancer subtype distribution. The study was performed in the same setting as the training.

#### Study Demographic and Patient Characteristics

This study was approved by each participating medical center institutional review board (IRB) ethics committee (In Israel: Sheba Tel-Hashomer, Rambam Health Care Campus, Shamir Medical Center (Asaf Harofe), Tzafon Medical Center (Poriya), and in S. Korea: Ewha Womans University Seoul Hospital).

Lung cancer patients and healthy individuals were recruited from April 2021 until January 2023 by the participating medical centers. All participants provided written informed consent in accordance with the provisions of the IRB in each medical center. Positive samples were considered as such if a chest computed tomography (CT) report indicated findings of lung cancer and was confirmed by an operative specimen or a relevant biopsy taken from the subject, as assessed by the attending oncologist or surgeon. Both tests were performed prior to the initiation of any anticancer treatment. This study included a total of 315 urine samples, out of which 165 samples were of patients who were diagnosed with lung cancer using LDCT or CT and pathology results, and 150 samples of healthy patients diagnosed negative for lung cancer. All healthy control patients went through chest LDCT or CT.

Demographics of the patients and healthy individuals are provided in Table 1. Sixty-five percent (65%) of the positive samples are of patients that were diagnosed at stages I and II (107 samples). Urine samples of five subtypes of lung cancer were included in this study: adenocarcinoma (128), large-cell carcinoma (6), squamous cell carcinoma (23), small-cell carcinoma (5), other subtypes of non-small-cell lung cancer (2), one blank and one unknown sample (Table 1). Individuals were excluded from the study if they had received preoperative chemotherapy or radiotherapy or any biological treatment.

As can be seen, there were pulmonary findings in the healthy control group as well. Thirty-seven percent (37%) of the subjects were diagnosed with some lesions using CT. In addition, in this group, there were 24 subjects who had previously diagnosed cancer of another origin as shown in Table 2.

Since some external factors may affect the sensing of VOC in urine by the BS, we also collected information about the smoking status and medications for the participant. Data shown in Table 3.

As expected, the proportions of smokers and medicated subjects were higher in diagnosed LC patients compared to the healthy controls. 

### 3.2. Validation Study Results

The results showed 154 samples as true positive and 137 samples as true negative. The PPV and NPV were 92% and 93%, respectively. The sensitivity was 93% and specificity was 91%, as detailed in Table 4.

This study confirmed that samples from patients diagnosed with lung cancer can be distinguished from samples provided by healthy subjects using the automatic BSP test. Blinded analysis resulted in high accuracy of 93% of early stages (I and II) of lung cancer. Moreover, when analyzing CT-reported findings from healthy subjects (Table 1), it is worth noting that none of the lesions affected the BSs’ performance. Findings such as soft tissue, granuloma, nodules, fibrosis, etc., reported by the radiologists in 55 out of the 150 healthy subjects were mostly regarded as “background” by the BSs. In addition, smoking and medication status (Table 3) did not affect the binary assessment results, with the BSs consistently providing the correct diagnosis of LC, and establishing the robustness of the test. These results are also independent of lung cancer subtype and stage.

### 3.3. Urine Sample Assessment Repeatability Testing after Sample Thawing and Refreezing

Previous studies have shown that urine forms precipitate during freeze/thaw cycles that depletes both calcium and proteins during storage at −20 °C for 12 h [31]. For validation of the freeze/thaw cycle accuracy, we tested the impact of multiple freeze/thaw cycles on the performance of the BSP. As we anticipate that the system will eventually be employed in a central lab setting, where samples are collected and mobilized from remote locations, in some cases, its capacity to perform adequately with samples that may have been thawed is important. 

For this validation study, we randomly selected 23 urine samples of both healthy and lung cancer-positive subjects from the Early Labs sample collection. The samples were used to validate the ability of the platform’s BSs to demonstrate repeated similar binary results for the chosen samples following repeated cycles of thawing and freezing of the same samples. Table 5 summarizes the sample population characteristics.

As shown in Table 6, ten (10) samples were taken from lung cancer patients and thirteen (13) from healthy subjects. Samples were assessed multiple times (on average, N= 6–9 times each, range 1–14) on various dates during a period of 64 days, by 3 independent BSs (BS 1–3). The exposure time of the sample in the system was between 1 and 5 s for each sample. Table 6 details the average success rate of the BSs, per sample.

From this study, it can be concluded that samples can be thawed and refrozen for a period of at least 9 weeks while remaining lung cancer-detectable. The detection for a sample by a BS is stable and repeatable. All samples had a high average detection success rate of at least 71%, as seen in Table 6.

### 3.4. The Utility of the BSP for Pooled Sample Diagnosis

The pooling method refers to the practice of combining biological samples from multiple individuals and testing them together as a single batch. The purpose of pooling is to increase testing efficiency and reduce the overall cost of testing, particularly in situations where the prevalence of the disease is low and most of the samples are expected to test negative.

**Figure 5:**
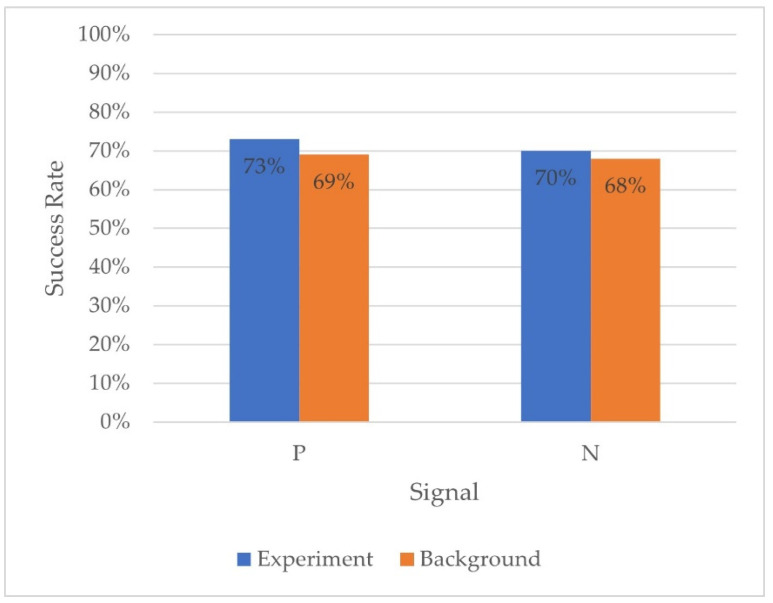
Success rates of BSs in diagnosis of pooled samples. Positive (P) and negative (N) pooled samples were randomly tested in a regular BSP session against the other samples (background).

To test the feasibility of the pooling method using our platform, we defined a total of 10 samples combined together to see if we could identify the positive sample when diluted into 9 other negative samples. We aimed to have an initial validation of the method in our BSP and to define its limits (Figure 5).

Exposure to the BSs was carried out as part of the daily session. Every session included two pools—four tubes (two control tubes and two test tubes). The tubes were embedded into the session: The first pair of tubes (control and test) was placed in the first third (position 1–16) of the session while the second was placed in the second third (position 17–32). There was a gap of at least three positions between one tube and another. Every tube was presented a single time to each biosensor in a session of 48 repeats. Nine BSs participated in the experiment.

The average difference between positive and negative experiment samples for each paired sample is not significantly different from 0 (−5%, SE ± 1, Wilcoxon test: *p* > 0.05).

This result demonstrates the ability of the BSP to correctly diagnose LC in 1/10 pooled positive samples to a level essentially similar to non-pooled samples. 

## 4. Discussion

The immediate need for inexpensive and noninvasive technology that would allow accessible, accurate out-of-hospital early detection of lung cancer has become vital. Early diagnosis is essential to achieving favorable outcome and prognosis in lung cancer. Unfortunately, the lack of alarming clinical signs in early stages coupled with the lack of efficient lung cancer screening programs results in diagnosis at later stages. As a result, currently, most patients are diagnosed too late and the delay in their diagnosis has significant implications for the patient’s 5-year survival chances. The faster a clinical team decides on the appropriate course of treatment, the better the patient’s prognosis. 

The Early Labs BSP system uses the animal’s unique, proficient, and accurate ability to smell lung cancer VOCs. VOCs reflect alterations in the pathophysiology and body metabolism processes, which have been studied in various types of cancers [32]. Cancer-associated VOCs are released from the affected tissue to feces or blood circulation by which the VOCs are exhaled in breath or excreted in urine [25]. Despite the rapid development of breathomics in the last four decades, no consistent, robust, and validated VOC signature profile for lung cancer has been identified [33].

Uchida et al. (2003) measured the relationship between the speed and accuracy of olfactory discrimination in rats. They found that speed of discrimination was independent of odor similarity, as measured by overlap of glomerular activity patterns of the odor receptors in the olfactory bulb. Even when pushed to psychophysical limits using mixtures of two odors, rats needed to take only one sniff (<200 ms at theta frequency) to make a decision of maximum accuracy. These results show that, for the purpose of odor quality discrimination, a fully refined olfactory sensory representation can emerge within a single sensorimotor or theta cycle, suggesting that each sniff can be considered a snapshot of the olfactory world [34]. The advantage of using rats is them being small and quick learners [34,35] with a natural sensory feature that can be employed to build layers of technology around it. Reports of first cases of cancer patients being diagnosed by animals (mainly dogs) appeared in 1989 and multiplied over the following years. In addition, interesting reports have emerged stating that cancers such as melanoma and bladder can be identified by the odor emanating from the source of the disease [33]. Functional studies have overcome many of the technical difficulties of controlling vapor stimuli and demonstrate that, with odor cues, rats display highly efficient learning rivaling that of primates. Rodents use urinary scent marks for communication with individual conspecifics in many social contexts. Human urinary scent involves genetic information such as species, sex, and individual identity as well as metabolic information [36]. Evolved odor signals involve some components that are genetically determined and not susceptible to disruption by metabolic and environmental influences. However, environmental factors, such as food type, bacterial gut flora, and social stress, as well as parasites status, also induce changes in volatile odors [36].

To the best of our knowledge, this is the first manuscript presenting a platform able to detect VOCs of lung cancer in a single urine test. The advantages of the test are (1) very high accuracy as demonstrated by the current double-blind study results showing 93% sensitivity and 91% specificity (Table 4); (2) rapid turnover of evaluation and provision of the results enabling patient management without unnecessary delays or flow disruption. The BSP can be used outside specialist settings and thus can considerably lessen the burden on health workload and costs. This study also demonstrated that the stability of urine containing lung cancer VOCs. Furthermore, samples maintained their VOC content stability in a frozen state, for at least 9 weeks, increasing the flexibility in sample management and logistics. In support of the latter result, previous studies showed that urine VOCs can be stable for over a year [37]. Early Labs has developed a new method of positive reinforcement training of animals within a technological environment, including collecting data from training sessions, to monitor the BSP process and increase the test’s accuracy. 

In order to avoid cross-sectional bias, we analyzed urine samples in respect to environmental factors that could have an impact on the test outcomes, namely, smoking and medications (Table 3). Since nicotine and cotinine are present in the urine of smokers, and smoking is a risk factor in this population, it was hypothesized that the test results may be hindered in smoking subjects. This was indeed shown relevant in a study of 40 LC patients using exhaled VOCs and using gas chromatography for diagnosis [32]. Similarly, many common drugs are not eliminated via the urinary system and may alter or mask lung cancer VOC (beta blockers, diuretics, certain antibiotics, certain NSAIDs). It was shown that when presented to the BSs as unknown samples, the binary diagnosis returned identical results with 93% sensitivity and 91% specificity, regardless of the smoking and medication status in both positively and negatively diagnosed patients. Thus, the BSP is indifferent to such environmental factors and diagnosis remains equally reliable.

Trained animals offer several advantages over instruments and equipment. The most significant feature is their sensitivity to low compound concentrations and their detection ability. Cost-effective solutions compared to sophisticated instrumentation and the ability to create an automated machine can also be a factor. Trained animals, in an automated platform, also have some limitations. They require ongoing training and additional care. Their performance can vary based on factors such as fatigue, distractions, or individual capabilities. The BSP results are based on three different independent BSs to neutralize the disadvantage.

A pool-testing approach can shorten the testing time and increase the test rate when a rapid diagnosis and treatment initiation is critical for achieving better outcomes. Such strategies have long been implemented, mostly for infectious diseases such as hepatitis B, C, HIV, and COVID-19 [38,39] with proven success. Our initial results demonstrate the utility of urine sample pooling in lung cancer patients as a proof of concept in 1:9 ratio. This result further establishes the accuracy of BSP and suggests that the platform may be useful in screening schemes such as in groups of individuals with high exposure to certain carcinogens, work hazard screening, heavy smokers, or in the general population. The exact algorithm for obtaining the optimal results from screening, such as sample volumes, dilution, and pooling time, should be further explored.

The landscape of lung cancer diagnostics has been changing with a shift towards less invasive and more rapid molecular and cellular assays such as liquid biopsy using RT-PCR from plasma, pleural effusion of bronchoalveolar lavage (BLA), circulating tumor cells, and DNA, alongside advanced imaging techniques such as endobronchial ultrasound with transbronchial needle aspiration (EBUS-TNBA), navigation bronchoscopy, and 18F-FDG PET/CT [40]. The benefits of these advanced methods are variable, and the costs are still high. While these are constantly improving, our BSP already presents a novel ancillary method to the conventional and developing tools, with high diagnostic value and good cost-effectiveness, good applicability, robustness, repetitiveness, consistency, and which is indifferent to environmental noise and does not require patient presence or invasive procedures and can be provided in a central lab setting. These features make the BSP an attractive candidate for further development and additional screening tool that can be obtained easily prior to LDCT for the selected population at risk.

## 5. Conclusions

This paper offers an illuminating clinical platform to deal with the pressing need for an early lung cancer screening and detection test tool. A combination of knowledge in zoology, biology, and technology has made the realization of this tool feasible. The BSP test is safe, short, objective, and can be performed repetitively—enabling periodic cancer monitoring as well as an aid to existing diagnostic methods. By using the BSP test, lung cancer can be screened and detected even at asymptomatic earlier stages and offer early diagnosis and treatment with hope for better survival rates. It is designed and proven to be lung cancer-specific, is indifferent to potential environmental masking, such as smoking and medication status, does not require subject participation, is noninvasive, inexpensive, accurate, and can be made available and accessible to all. The BSP was shown to provide PPV and NPV values of 92%, which can be considered on the high end for experimental urinary tests for several types of cancer, such as bladder [41], cervical [42], and lung [43], thus positioning this method as an attractive option for future diagnosis, screening, and even postoperative follow-up of patients treated for lung cancer with the hope for better outcomes and survival.

## Figures and Tables

**Table 1:** Urine sample subjects’ characteristics.

Lung Cancer-Diagnosed Group = Positive Samples (N = 165)		
Gender	F	79
	M	86
Mean Age		68.19
Stage 0 (1)		1
Adenocarcinoma		
Stage 1 (92)		
Adenocarcinoma		77
Large-cell		1
Squamous cell		13
Other non-small-cell		1
Stage 2 (14)		
Adenocarcinoma		5
Squamous cell		4
Large-cell		3
Small-cell		2
Stage 3 (16)		
Adenocarcinoma		15
Other non-small-cell		1
Stage 4 (40)		
Adenocarcinoma		29
Large-cell		2
Squamous cell		6
Small-cell		3
**Healthy Group = Negative Samples (N = 150)**		
Gender	F	51
	M	99
Mean Age		51.22
LDCT Main Radiologic Findings (one or more)		55 (37%)
Soft tissue		20 (36%)
Granuloma		12 (22%)
Fibrosis		8 (14.5%)
Nodules		42 (76.3%)

**Table 2:** Cases of other cancer types in the LC healthy control group.

Cancer Type	Number of Patients
Appendix neoplasm	1
Biliary tract cancer	1
Skin cancer	1
Breast cancer	8
Esophageal cancer	2
Rib neoplasm	1
Pancreatic carcinoma	5
Peritoneal mesothelioma	1
Solitary fibrous tumor of the pleura	1
Thymoma	3
Total	24

**Table 3:** Environmental factor distribution among participants.

Smoking Status	Healthy Controls	LC Patients	Total
No	32 (21%)	50 (30%)	82
Yes	28 (19%)	34 (21%)	62
Unknown	90	81	171
Total	150	165	315
Medications			
No	34 (23%)	15 (9%)	49
Yes	26 (17%)	62 (38%)	88
Unknown	90	88	178
Total	150	165	315

**Table 4:** Validation study results.

Positive	Samples	Negative	Samples
True positive	154	False positive	13 ^2^
False negative	11 ^1^	True negative	137
Total	165	Total	150
Sensitivity	Specificity	PPV	NPV
93%	91%	92%	93%

Sample data: ^1^ false negative (11 samples) combining 11 (5, 2, 4 from stage I, III, IV, respectively); ^2^ false positive (13 samples): 4 of the samples have one or more lesions.

**Table 5:** Urine samples characteristics.

	Lung Cancer		Healthy
Gender	2 males, 8 females		11 males, 2 females
Age	65.6 ± 10.7		50.6 ± 9.4
Cancer stage	I-IIIA, I-IIB		N/A
Cancer subtypes	Adenocarcinoma	8	N/A
	Squamous cell carcinoma	1	N/A
	Small-cell carcinoma	1	N/A

**Table 6:** Urine sample diagnosis success rates after six freeze/thaw cycles for each sample.

		Sample Name	Avg. ^1^ SR ± STD
Lung cancer patients		LC1	97% ± 2%
		LC2	73% ± 11%
		LC3	98% ± 1%
		LC4	80% ± 20%
		LC5	87% ± 13%
		LC6	80% ± 14%
		LC7	71% ± 8%
		LC8	81% ± 15%
		LC9	97% ± 2%
		LC10	80% ± 14%
Healthy patients		H1	95% ± 4%
		H2	93% ± 5%
		H3	100% ± 0%
		H4	93% ± 7%
		H5	98% ± 2%
		H6	88% ± 10%
		H7	84% ± 5%
		H8	87% ± 7%
		H9	77% ± 16%
		H10	84% ± 17%
		H11	72% ± 10%
		H12	94% ± 5%
		H13	74% ± 13%

^1^ The right column presents the averaged success rates (%) of N exposures on average, N = 6–9 times each, (min N = 1; max N = 14) on various dates during a period of 64 days to the same sample ± SD for all BSs.

## Data Availability

The data presented in this study are available on request from the corresponding author.
